# Neural Correlates of Reflection on Present and Past Selves in Autism Spectrum Disorder

**DOI:** 10.1007/s10803-018-3621-y

**Published:** 2018-06-05

**Authors:** Hanna B. Cygan, Artur Marchewka, Ilona Kotlewska, Anna Nowicka

**Affiliations:** 1 Laboratory of Social Psychology, Department of Ergonomics, Central Institute for Labour Protection - National Research Institute, 16 Czerniakowska Street, 00-701 Warsaw, Poland; 2 Laboratory of Psychophysiology, Department of Neurophysiology, Nencki Institute of Experimental Biology, Polish Academy of Sciences, 3 Pasteur Street, 02-093 Warsaw, Poland; 3 Laboratory of Brain Imaging, Neurobiology Center, Nencki Institute of Experimental Biology, Polish Academy of Sciences, 3 Pasteur Street, 02-093 Warsaw, Poland; 4 Faculty of Humanities, Nicolaus Copernicus University, Torun, Poland; 5 Department of Psychological and Brain Sciences, Dartmouth College, Hanover, NH USA

**Keywords:** fMRI, Self-referential processing, Self-continuity, Autobiographical memory

## Abstract

Previous studies indicate that autobiographical memory is impaired in individuals with autism spectrum disorder (ASD). Successful recollection of information referring to one’s own person requires the intact ability to re-activate representation of the past self. In the current fMRI study we investigated process of conscious reflection on the present self, the past self, and a close-other in the ASD and typically developing groups. Significant inter-group differences were found in the Past-Self condition. In individuals with ASD, reflection on the past self was associated with additional engagement of the posterior cingulate and posterior temporal structures. We hypothesize that this enhanced activation of widely distributed neural network reflects substantial difficulties in processes of reflection on one’s own person in the past.

## Introduction

One fundamental feature of the human conscious experience is a sense of self that persists across time (Gallagher 2000; Moran et al. 2006; Morin 2006). The sense of self-continuity is related to autobiographical memory and it is based on the ability to consolidate different and temporally separated pieces of self-related information into one coherent whole (McAdams 2001; Conway 2005). However, processing personal changes across the lifespan requires the ability to strictly distinguish between representations of the present and the past selves; this ability was hypothesized to be crucial for the formation of a stable identity during the late adolescence and early adulthood (McAdams 2001).

It is noteworthy that several studies on autism spectrum disorder (ASD) have reported difficulties in remembering the past (Bowler et al. 2007). Specifically, this was the case for autobiographical memory tasks that assessed the ability to recall personally experienced events and personal semantic facts (for review see: Brezis 2015). In children with ASD, both semantic and episodic autobiographical memory is reduced (Bruck et al. 2007; Bon et al. 2012; Goddard et al. 2014). By adulthood, individuals with ASD show a spared memory for semantic autobiographical memory, alongside reduced episodic autobiographical memory (Klein et al. 1999; Crane and Goddard 2008). Individuals with ASD consistently recollect not only fewer events from their past than matched control subjects but also take substantially longer to do so (e.g. Goddard et al. 2007; Crane and Goddard 2008; Crane et al. 2009; Lind and Bowler 2009; Adler et al. 2010).

It was proposed that autobiographical memory difficulties in this clinical group may be related to a reduction of cognitive resources for auto-noetic awareness, i.e. the conscious re-experiencing of past events (Tanweer et al. 2010; Crane et al. 2012). Such disturbed ability to recall the past can result in impaired anticipation of and planning for the future, which in turn may lead to the lack of flexibility and enhanced anxiety, that is typical for individuals with ASD (Kreslins et al. 2015). However, impaired autobiographical memory may also be viewed in the light of disturbed self-referential cognition in ASD (e.g. Crane et al. 2009; Lombardo and Baron-Cohen 2010; Glezerman 2013; Cygan et al. 2014; Nowicka et al. 2016). If difficulties in remembering the past and disrupted self-referential processing are typical for ASD (for review see: Lind 2010), one may expect that processing of the past self is atypical in this group. In the typically developing (TD) population, the neural underpinnings of processing temporally-distant selves have often been investigated using the self-reflection task, i.e. evaluation of whether some personality traits are suitable to describe one’s own person (D’Argembeau et al. 2008, 2010; Luo et al. 2010; Kotlewska and Nowicka 2016). On a behavioral level, a very recent study reported that teenagers with ASD presented reduction in the retrieval of personality traits, poorer knowledge about the self and others and impairment in mentalizing abilities (Robinson et al. 2017). In addition, a growing body of evidence indicates differences between ASD and TD groups in the self-referential attribution capacities (e.g. Williams 2010; Woods 2012). However, to the best of our knowledge, there is no study on individuals with ASD that has focused on the neural correlates associated with the process of reflection in reference to the present and past selves.

Accordingly, we designed a fMRI experiment aimed at verifying the hypothesis of an impaired process of attribution related to the past self in individuals with ASD. The two other targets of reflection were the present self and a close-other. Based on the numerous fMRI studies, reporting atypical self-referential processing reflected in altered activation of the temporoparietal junction (TPJ), superior temporal sulcus (STS), cingulate cortex (CC), medial prefrontal cortex (MPC), and precuneus (e.g. Lombardo et al. 2011; Kestemont et al. 2016), we expected to observe differences between individuals with ASD and control subjects in activations of the aforementioned brain regions during assignment of trait adjectives to one’s own person in the past.

## Methods

### Subjects

Fifteen young males with ASD and 15 control subjects participated in this study. The clinical diagnosis of individuals with ASD was confirmed using the Polish translation of the Autism Diagnostic Observation Schedule—ADOS (module 4) (Lord et al. 2008). The ASD group was recruited by the psychologists and therapists from the SYNAPSIS foundation which provides diagnosis assistance and therapy for people with ASD and their families.

Control subjects were matched one-to-one to individuals with ASD in terms of age, sex, handedness, and IQ-score. Subjects’ IQs were evaluated using Polish version of the Wechsler Intelligence Scale for Adults—Revised (WAIS-R, PL) (Brzeziński et al. 2004). The maximal IQ difference between each individual with ASD and the matched control subject was ± 15 (see Table 1). The maximal age difference between each individual with ASD and the matched control subject was ± 8 months. In the ASD group, the mean age was 24 years and 4 months. In the control group, the mean age was 24 years and 2 months. Results of statistical comparisons (both independent- and paired-samples t-tests) of the age and IQ (full, verbal, non-verbal) in the two groups (ASD and TD) are included in description of Table 1.

**Table 1:** Characteristics of the ASD and TD groups

ASD							TD				
Subject	Age	IQ			ADOS		Subject	Age	IQ		
		Full	Verb	Non-verb	Communic	Social Int			Full	Verb	Non-verb
A1	22:4	106	109	103	3	6	C1	22:1	116	130	97
A2	21:6	106	116	93	3	8	C2	22:3	119	124	110
A3	22:9	97	108	83	6	6	C3	22:2	105	108	103
A4	21:11	117	125	107	3	7	C4	21:11	128	130	123
A5	21:7	102	96	109	5	8	C5	21:7	89	86	93
A6	25:5	108	97	121	4	2	C6	25:9	121	116	126
A7	27:2	111	124	93	2	5	C7	26:10	118	111	126
A8	26:3	86	100	69	3	3	C8	26	97	99	93
A9	22:2	93	96	90	5	8	C9	22:9	101	99	104
A10	27:4	128	143	107	3	7	C10	26:7	132	139	122
A11	24:1	116	114	118	3	3	C11	24:7	127	126	119
A12	28:1	108	112	101	8	4	C12	28:4	110	107	114
A13	27:3	115	116	109	3	5	C13	27:8	123	123	117
A14	24:2	105	110	97	5	8	C14	24:10	115	131	122
A15	21:3	95	100	90	3	9	C15	21:8	110	123	97

Age (years:months), IQ scores for both groups (full—full scale, verb—verbal scale, non-verb—non-verbal performance scale), and ADOS scores for individuals with ASD (communic—communication, social int—social interaction)

The independent-samples t-test indicated that age difference was non-significant (*P* = 0.897). In the case of IQ, between-group difference reached the level of statistical significance (*P* = 0.043). It turned out that this effect was driven by IQ differences in non-verbal IQ (*P* = 0.019) and not the verbal one (*P* = 0.268). The paired-sample t-test also indicated no significant group differences in age (*P* = 0.350). In the case of IQ levels, between-group difference was significant in reference to the full scale (*P* = 0.001) and the non-verbal scale (*P* = 0.003). The between-group difference in verbal IQ scale did not reach the level of statistical significance, however, a weak trend was found (*P* = 0.076). In the light of verbal demands of our behavioral task, a lack of differences between the ASD and control group in verbal IQ may support our opinion that between-group differences in fMRI findings were not related to differences in verbal IQ

All subjects had normal or corrected-to-normal vision and did not take any medication at the time of the experiment. Subjects were financially compensated for their participation.

The study conforms to the World Medical Association Declaration of Helsinki. The experiment was undertaken with the understanding and written consent of each subject, and the experimental protocol was approved by the local Ethics Committee (University of Social Sciences and Humanities, Warsaw, Poland).

### MRI Acquisition

MRI data acquisition took place at the Laboratory of Brain Imaging, Neurobiology Center, Nencki Institute of Experimental Biology on a 3-Tesla MR scanner (Siemens Magnetom Trio TIM, Erlangen, Germany) equipped with a 32-channel phased array head coil.

Functional data were acquired using a T2*-weighted gradient echo echo-planar imaging (EPI) sequence with the following parameters: time repetition = 2190 ms, time echo = 30 ms, flip angle = 90°, in plane resolution = 64 × 64 mm, field of view = 192 mm, and 33 axial slices with 3.6 mm slice thickness with no gap between slices. Detailed anatomical data of the brain were acquired with a T1-weighted (T1w) MP-RAGE (time repetition = 2530 ms, time echo = 3.32 ms) sequence. Head movements were minimized with cushions placed around the subjects’ heads.

### Stimuli and Experimental Design

The experimental procedure was prepared in Presentation® software (Neurobehavioral Systems, Inc., Albany, CA). Stimuli were presented centrally on a 21″ MR-compatible LCD screen located in the back of the MR room. Subjects viewed the stimuli through an angled mirror attached to the head coil.

The set of stimuli consisted of adjectives referring to personal characteristics (half positive, half negative), the majority of which were selected from Anderson’s list (Anderson 1968) and translated into Polish. Adjectives were written in white capital letters against a gray background.

The experimental paradigm used a mixed block and event related design. A mixed-block design allowed to separate experimental conditions as defined by the target of reflection/evaluation: Present-Self, Past-Self, Close-Other. Within each block, in turn, event-related design was used. Using event-related design in each block enabled us to select trials with ‘yes’ responses. This was important as we were interested in separating trials with attributes judged as suitable to describe a given target of reflection/evaluation (present-self, past-self, close-other). There is no doubt that the self-referential processing occurred in trials with ‘yes’ responses as confirmatory responses provided a very clear indication that participants were confident about their personal characteristics in the specified time-period (at present, in the past). A similar approach (i.e. splitting the data based on whether adjectives were judged as self-descriptive or non-self-descriptive) was used in some previous studies (e.g. Zhang et al. 2013).

Each block consisted of 72 events/trials during which adjectives describing personal features were presented. In each trial an adjective was displayed for 3.5 s, followed by a fixation cross presented for 1 s. Adjectives were presented in pseudo-randomized order. In each condition, different sets of adjectives were presented. The assignment of lists to experimental conditions was counter-balanced on the group level.

A detailed instruction was applied prior to each block to help the subjects enter a state of reflection about the particular person (present-self, past-self, close-other). Additionally, one of the question was presented between sub-sets of 24 stimuli: “Are you…”; “When you were about 14, were you…”; “Is your father (/brother/friend)…”. Subjects were tasked with judging whether a given adjective was suitable to describe/characterize a person specified in the instruction in a single block (present-self, past-self, close-other). Yes/no responses were given by pressing one of two buttons on a response pad. In the subsequent analyses of behavioral and fMRI data only trials with ‘yes’ responses were included (the ‘no’ responses would add some ‘noise’ to the analyzed BOLD signal). Each block lasted approximately 6 min.

Participants were asked to freely choose their Close-Other among the persons who were the most significant to the participant ‘at present’, i.e. at the time of our experiment, with the only restriction: the gender should be the same as the gender of our participants, i.e. male. This was done to avoid a situation in which a pre-defined person is not really close to a particular subject; and also we wanted to avoid different grammatical forms of adjectives used to describe females in Polish. Thus, prior to the fMRI study participants were requested to assign a closely-related person, and describe their relationship briefly. Only 3 control subjects chose their close-friend and a vast majority of the subjects (27) chose their father or brother (ASD group: 12—father; 3—brother; control group: 7—father; 5—brother).

Moreover, the past self was defined as ‘the self in middle high school.’ Therefore, in the past-self condition our subjects were asked to ‘mentally move’ to the time when they attended middle high school. As our subjects were about 24 years old, such ‘past self’ seemed to be well-defined and appropriate for investigation of the self across time (D’Argembeau et al. 2008, 2010; Kotlewska and Nowicka 2016).

Before the experimental MRI session, each subject took part in a training session in a mock MRI scanner situated in the Laboratory of Brain Imaging. The training session was performed between 1 week and 1 day prior the experimental session. During the training session shorter stimuli sets were used, with different stimuli sequences and different assignment of adjectives lists to experimental conditions. The training session aimed at familiarizing ASD subjects with the MRI environment. Control subjects underwent the same training procedure in order to equalize the experimental experience of both groups.

### fMRI Data Analysis

Statistical Parametric Mapping (SPM12b, Wellcome Trust Center for Neuroimaging, London, UK) running on MATLAB R2013b (The Math-Works Inc. Natick, MA, USA) was used for data preprocessing and statistical analyses. First, functional images were motion corrected (head movements were < 4 mm in all cases, with no significant differences in movements between groups). Then, structural images from single subjects were co-registered to the mean functional image. The functional images were normalized to MNI space using compositions of flow fields and group-specific template to a 2 mm isotropic voxel size. Finally, the normalized functional images were smoothed with 6 mm isotropic Gaussian kernel.

In the first-level of statistical analysis, all experimental conditions and head movement (translation and rotation) parameters were entered into the design matrix. The data were modeled for each of the three experimental fMRI runs and using the canonical hemodynamic response function convolved with the experimental conditions (present-self, past-self, close-other). The model included only the events that were positively categorized (i.e. ‘yes’ responses were given) to a relevant experimental condition separately by each subject.

The following single *t*-tests were computed at 1st level (within subject): present-self, past-self, and close-other. In the 2nd level (between subject) analysis, a flexible factorial design was used with intergroup factor ASD vs. control and within group factor ‘condition’ (present-self vs. past-self vs. close-other). Additionally, for the all-subject analysis (ASD + TD) a full factorial design was used for statistical calculation of the within-group factor ‘condition’: present-self—fix (fixation cross) vs. past-self—fix vs. close-other—fix.

On a group level a voxel-wise height threshold of *P* < 0.001 (uncorrected) combined with a cluster-level extent threshold of *P* < 0.05 (cluster size > 30 voxels; corrected for multiple comparisons using the family wise error (FWE) rate) was employed for whole brain analyses.

MNI coordinates were translated to Talairach space using GingerALE software (http://www.brainmap.org). TalairachClient 2.4.2 was then used to identify the activated structures (Lancaster et al. 2000; http://www.talairach.org).

## Results

### Behavioral Results

Table 2 presents the mean raw numbers and mean relative percentages of positive and negative adjectives assigned to each experimental condition: present-self, past-self, close-other by individuals with ASD and control subjects. The number of omitted trials was very low in each group (mean number: 1.4 and 0.36 for ‘present-self’, 1.0 and 0.16 for ‘past-self’, and 1.13 0.2 for ‘close-other’, in the ASD and control group, respectively). It did not differ neither between groups nor conditions (all *P*s > 0.290).

**Table 2:** Behavioral results: mean raw numbers, mean relative percentages and their standard deviations (in brackets) of positive and negative adjectives assigned by ASD and TD groups to each experimental condition: present-self, past-self, close-other

Condition	Positive adjectives assignments		Negative adjectives assignments	
	ASD	TD	ASD	TD
Present-self	23.5 (7.1) 65.3% (19.7)	23.6 (5.3) 65.6% (14.7)	12.0 (5.8) 33.3% (16.1)	10.1 (3.8) 28.1% (10.6)
Past-self	18.6 (5.2) 51.7% (14.4)	22.0 (4.5) 61.1% (12.5)	14.6 (8.8) 40.6% (24.4)	12.7 (6.6) 35.3% (18.3)
Close-other	24.4 (5.7) 67.8% (15.8)	23.3 (3.3) 65.6% (9.2)	7.9 (6.3) 21.9% (17.5)	9.6 (5.0) 26.7% (13.9)

Analysis of ‘yes’ responses with group and experimental condition as factors revealed no significant main effects or interactions. However, between-group statistical comparisons—performed separately for each experimental condition—for the number of positive and negative attributes assigned by individuals with ASD and control subjects showed that in the ‘past-self’ condition, significantly more positive attributes were ascribed by control subjects than by individuals with ASD (*F*_1,28_ = 4.462; *P* = 0.044). No significant effects were found in the ‘present-self’ and ‘close-other’ conditions (see Table 2).

### fMRI Results

#### Effects for ASD + TD Subjects

**Fig. 1:**
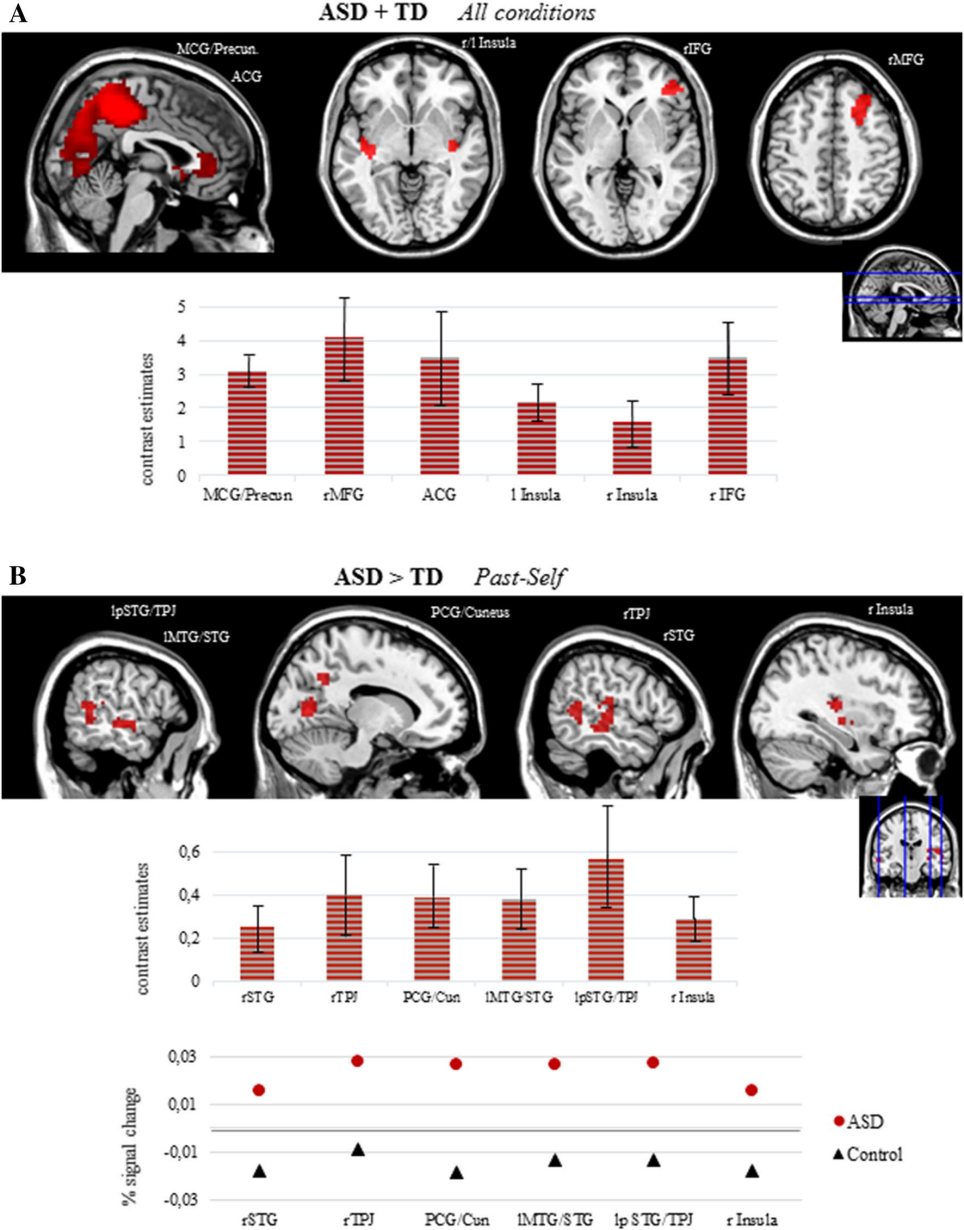
Results of fMRI analysis. **a** Results of contrast analysis for all participants (ASD + TD) and all conditions (‘self’ + ‘past-self’ + ‘close-other’) together. The graph placed below brain images present mean values and standard deviations of group-level contrast estimates for each of the clusters of significant activity. **b** Results of between group comparison (ASD > TD) for ‘past-self’ condition. In both panels regions of significant contrasts (FWE-corrected at the cluster level, *P* < 0.001; cluster size > 30 voxels) are plotted (red color) on the template of grey matter tissue probability map (TMP.nii, SPM12). The upper graph placed below brain images presents mean values and standard deviations of group-level contrast estimates for each of the clusters of significant activity. Lower graph presents mean values of percent signal change for each of the clusters of significant difference in activity between ASD and TD group. *r* right, *l* left, *p* posterior, *MCG* middle cingulate gyrus, *Precun*. precuneus, *MFG* middle frontal gyrus, *ACG* anterior cingulate gyrus, *IFG* inferior frontal gyrus, *STG* superior temporal gyrus, *TPJ* temporoparietal junction, *PCG* posterior cingulate gyrus, *MTG* middle temporal gyrus. (Color figure online)

Analyses for the two groups collapsed together (ASD + TD group) revealed main effect of task with clusters of significant activity for all analyzed contrasts (‘present-self—fix’, ‘past-self—fix’, ‘close-other—fix’). For all conditions taken together we found a number of cortical structures that revealed positive contrast values (see Table 3; Fig. 1a), including: the middle cingulate gyrus—MCG/precuneus (*T* = 12.94; 4187 voxels), right middle frontal gyrus—MFG (*T* = 6.13; 169 voxels), anterior cingulate gyrus—ACG (*T* = 4.66; 109 voxels), left insula (*T* = 6.5; 237 voxels), right insula (*T* = 5.70; 87 voxels) and right inferior frontal gyrus—IFG (*T* = 4.9; 56 voxels). However, there were no significant differences between specified contrasts.

**Table 3:** fMRI results: significant activations with peak Talairach coordinates and *P*-values

Structure	Group/condition	Peak coordinates x, y, z	*P* (FWE-corrected)
MCG/precuneus	ASD + TD/all conditions	12, − 39, 42	0.0001
Right MFG		29, 28, 49	0.0001
ACG		5, 42, 0	0.0001
Left insula		− 38, − 18, − 4	0.0001
Right insula		36, − 4, − 14	0.002
Right IFG		40, 39, 14	0.01
Right STG	ASD > TD/all conditions	50, − 21, 14	0.029
Right STG	ASD > TD/‘past-self’	54, − 21, 0	0.0001
Right TPJ		54, − 49, 7	0.047
PCG/left cuneus		− 13, − 70, 7	0.0001
Left MTG/STG		− 59, − 14, − 4	0.0001
Left posterior STG/TPJ		− 59, − 46, 11	0.007
Right insula		33, − 21, 14	0.0001

*ASD* Autism Spectrum Disorder group, *TD* typically developing group, *MCG* middle cingulate gyrus, *MFG* middle frontal gyrus, *ACG* anterior cingulate gyrus, *IFG* inferior frontal gyrus, *STG* superior temporal gyrus, *TPJ* temporoparietal junction, *PCG* posterior cingulate gyrus, *MTG* middle temporal gyrus

#### Effects for the ASD Group vs. the TD Group

First, for all experimental conditions (present-self, past-self, close-other) collapsed together, we found positive differences between ASD and TD groups in contrast estimates in response to adjectives that were selected by the subjects as descriptive for each of the specified conditions. Significantly stronger activations in the ASD group were observed in one structure—the right posterior superior temporal gyrus—STG (*T* = 5.08; 30 voxels). Interestingly, the additional analysis revealed that the cluster of enhanced activation in ASD subjects was wider when the ‘present-self’ condition was excluded from the analysis. The comparison of ‘past-self’ and ‘close-other’ taken together revealed larger cluster of increased activity in the ASD group in the same region of STG/TPJ (*T* = 5.14; 107 voxels). We found no regions of significantly stronger activity in TD group vs. ASD group.

Secondly, inter-group comparisons were done for each experimental condition (present-self, past-self, close-other). Significant clusters of enhanced activation in individuals with ASD were found only in the case of the past-self condition. Such atypical activity was observed in right insula and right STG (*T* = 4.70; 177 voxels), right TPJ (*T* = 4.26; 38 voxels), posterior cingulate—PCG/cuneus (*T* = 4.54; 344 voxels), left middle temporal gyrus—MTG/STG (*T* = 4.39; 102 voxels) and left posterior STG/TPJ (*T* = 4.27; 63 voxels) (see Table 3; Fig. 1b). For all analyzed experimental conditions we found no cortical regions of significantly stronger activity in TD vs. ASD group.

## Discussion

A critical aspect of self-related cognition is the ability to remember events that occurred in one’s past (McAdams 2001; Conway 2005). It has been suggested that difficulties in accessing specific autobiographical memories in ASD may be due to problems in using the self as an effective memory organizational system (Crane et al. 2009). In a study examining narratives of self-defining and everyday autobiographical memories in adults with ASD, it was shown that individuals with ASD extracted less meaning from their memories than adult controls, which may be interpreted as a failure in using past experiences to update the self (Crane et al. 2010). However, no previous studies have examined the temporally-distant selves in ASD using a task other than memory tasks.

In the present fMRI study, we investigated the neural correlates of adjectives (referring to personal features) assignment to the present self, the past self, and a personally-relevant person in high functioning individuals with ASD. During fMRI scanning subjects were required to evaluate whether adjectives were suitable to describe their person at present, their person in the past (at the time of middle high school), and a close-other (friend or family member). The set of adjectives for each target of reflection included an equalized number of positive and negative features.

On the behavioral level, no significant between group effects were found while analyzing all experimental conditions together (present-self, past-self, close-other). However, analyses done for each condition separately revealed significant inter-group differences in the number of positive attributions made while evaluating personal features of the past self. In this case, individuals with ASD presented significantly weaker positivity bias (Mezulis et al. 2004) than the TD group. On the other hand, both groups presented similar positivity bias in the case of present self and the close-other conditions.

On the neural level, fMRI results for both groups of subjects collapsed together showed significant activations in MCG, ACG, Insula, and MFG, typically reported for self-related information processing and mentalizing (e.g. Lombardo et al. 2011; Murray et al. 2012; Kestemont et al. 2015) as well as for reflection on present and past selves (D’Argembeau et al. 2008). In addition, we found activations in the right IFG and precuneus that were previously reported specifically in the case of self-reflection across time (D’Argembeau et al. 2008). However, we did not found significant differences in brain activation between self-related and other person-related conditions. It is worth noting that in the current study, we compared brain activity during evaluation of the self with the close-other—not like in many of the previous studies with a famous (e.g. Kelley et al. 2002) or familiar but not personally known person (Pfeifer et al. 2007, 2013a). As it has been already shown, this distinction is important for the extent of differences between brain responses for the self and other. The areas of activity are highly common if the other is personally known, very significant person (Kennedy and Courchesne 2008; Tacikowski et al. 2013; Laurita et al. 2017). A lack of differentiation between the self and the other in cortical midline structures (CMS) has been observed in prior studies, most frequently when targets of reflection were either very close or similar to the self (Ochsner et al. 2005; Krienen et al. 2010). Interestingly, results of one of the studies on self-processing, where close friend was included as a condition in an evaluative perspective task, revealed the differences in brain activity between self and other person conditions only in the group of adolescents and not in the group of adults (Jankowski et al. 2014). It is also worth noting that in our study the contrasts for conditions were made for both groups ASD and TD collapsed together. Therefore, the effects specific for ASD group may bias our comparisons resulting in less typical activation patterns that had not been reported in previous research on general population. Specifically, it was previously shown that in contrast to TD groups, CMS activations in individuals with ASD were similar for the self and other (e.g. Kennedy and Courchesne 2008) or even stronger for other than the self condition (Lombardo et al. 2010).

Moreover, analysis of fMRI data revealed differences between the ASD and TD groups that were common for all experimental conditions (present-self, past-self, close-other) collapsed together. During the attribution process ASD subjects exhibited elevated activity in one cortical structure—the right posterior STG/TPJ. Interestingly, further exploration of this result suggested that this stronger activity was driven mainly by the inter-group difference in two of the analyzed conditions—past-self and close-other. Finally, analyses done for each condition separately showed significantly stronger activation in individuals with ASD when compared to TD group in the ‘past-self’ condition in a number of brain regions: the right insula, PCG, right and left posterior STG/TPJ, and left MTG. Importantly, percent signal change measures suggest that the inter-group differences in all of those regions are driven by the enhanced activity among ASD subjects and diminished activity in TD group.

The effect observed in the ASD group during retrieval of personal attributes about the past self could not be directly related to previous findings, as none of the earlier fMRI studies on ASD investigated the neural mechanisms underlying reflection on personal features of the past self. Nevertheless, there are some important links between our results and the published literature. For example, in a very recent fMRI study that included subclinical and clinical ASD groups, subjects were engaged in a causal attribution task that included self, another person, and situation conditions. Similarly to our findings, hyperactivity in the neural network including the posterior STS and TPJ was reported in individuals with ASD (Kestemont et al. 2016). Our results are also in line with several previous studies that revealed stronger activity or diminished task selectivity of relevant brain structures during performance of mentalizing about self and others (e.g. Mason et al. 2008; Lombardo et al. 2011; Schulte-Rüther et al. 2011).

Importantly, we observed atypical processing of information related to the past self on both the behavioral and neural levels. Behaviorally individuals with ASD were less inclined than their TD counterparts to assign positive attributes to their own person in the past. It was previously shown that TD youths make more positive self-appraisals than youths with ASD during self-evaluation tasks (Pfeifer et al. 2013b). ASD children were also characterized by diminished preference for memorizing positive over negative adjectives when those attributes had to be referred to themselves (Burrows et al. 2017). Moreover, in our study the ASD group was characterized by broad activity mainly in the posterior brain regions while reflecting on the past self. Importantly this extended network of activity includes areas that were disengaged in the TD group in the past-self condition. These results reflect highly atypical pattern of brain regions involved in retrieval of autobiographical memory and self-characteristics from the past in individuals with ASD. We hypothesize that stronger activations in the past-self condition observed in individuals with ASD may reflect the higher—in comparison to control subjects—level of difficulty of reflecting on one’s own person in the past. This notion is based on numerous studies showing that the more difficult the task the stronger and/or larger the activations (e.g. Gould et al. 2003; Erickson et al. 2007; Nowicka et al. 2011).

In line with the latter, enhanced engagement of right and left posterior temporal regions and parts of the TPJ may suggest the need for involvement of some additional brain resources by subjects with ASD to process more ambiguous social information. It was previously proposed that hyperactivity of the STS/TPJ may reflect the engagement of those areas that are involved in a more general cognitive processing which compensate for impairments in attribution judgements (Kestemont et al. 2016). In our study, apart from the right TPJ—the structure consequently linked to mentalizing abilities—we found activity in neighboring areas of the STS/STG and MTG. In previous research, those structures were linked to recognition of other people’s intentions mainly based on their body and gaze movement, and had direct connections with the mentalizing networks (Pelphrey et al. 2011). Importantly, activations in TPJ and adjacent posterior STG, found in the ASD group, may be also linked to reflected self-appraisals—a task involving mentalizing processes (Pfeifer et al. 2009; Pfeifer and Peake 2012). Although our participants were not instructed to think about other people’s perspectives on the self, they seemed to activate components of the social perception network including TPJ and posterior STG (Pfeifer et al. 2009, 2017). It was previously shown that neuro-typical adolescents when compared to adults during both direct and reflected self-appraisals more extensively engage brain regions involved in perspective-taking, like TPJ (Pfeifer et al. 2009). This may suggest a delayed development of cognitive networks involved in the past self processing in examined adult ASD subjects. It may be also hypothesized that in individuals with ASD their characteristic of the past self is based to a higher extent on information received from social environment (e.g. family members, therapists) than on the internalized knowledge about themselves. The later notion, however, is highly speculative in nature.

Additionally, we found a cluster of increased activity in our ASD group that included the very posterior part of the cingulate gyrus and cuneus. Activity in this area may suggest the involvement of imagery and memory of concrete episodic events using visual representations during retrieval of past self attributes (e.g. Addis et al. 2004). This enhanced activation of posterior brain regions found in the ASD group may also indicate that individuals with ASD seem to be ‘visual thinkers’ (Grandin 2009), i.e. individuals with ASD may tend to visualize one selves in the past in order to be able to describe/characterize themselves in the past.

All in all, findings of the current study may suggest that in our study individuals with ASD recruited additional areas to engage imagination and more embodied representations of their own behaviors and intentions from the past, which were crucial for recollection of personal traits describing the past self. They may also refer to the information about their past characteristics received from their social environment to a higher extent than the TD adults. Thus, recruitment of additional areas may serve as a kind of a compensatory mechanism for abnormalities in autobiographic memory reported among subjects with ASD (Brezis 2015).

While significant inter-group differences were found in the past-self condition, they were absent in the present-self conditions. This may also be viewed in the light of research on autobiographical memory in ASD. Studies on episodic autobiographical memory for different periods in subjects’ lifetimes found that in ASD memories for recent events were more specific and detailed than for remote events (Bruck et al. 2007; Crane and Goddard 2008; Tanweer et al. 2010; Goddard et al. 2014). Thus, one may assume that assignment of adjectives to one’s own person at present was much easier than to the self in the past; the latter forced individuals with ASD to ‘mentally move’ to their far past in order to judge whether some traits were suitable to characterize their person while attending middle high school. There is rather no doubt that this task can be characterized as episodic autobiographical memory. However, one may speculate that depending on the trait presented to participants, their personal experience and their self-awareness, they may sometimes refer also to their semantic knowledge. The reason is that some of the personal characteristics are rather stable over time (e.g. intelligent, blond) and other not necessarily (e.g. responsible, tall). Therefore, we assume that semantic and episodic memory may be interactively engaged, if a subject could not retrieve his specific characteristic in the past and had to ‘mentally move’ to the past situations using episodic memory.

Our supposition is directly supported by fMRI findings of the present study, i.e. stronger activation of posterior brain regions, including the PCG and bilateral temporal areas in the ASD group. Importantly, numerous previous studies have linked those structures with both episodic and semantic autobiographic memory retrieval (for meta-analysis see: Martinelli et al. 2013). Martinelli et al. proposed to include the conceptual self as one of the components of autobiographic memory that specifically relates to abstract self-representation and personal traits identified and internalized across the lifespan experience. However, the conceptual self was specifically related to activity in the frontal brain structures (Martinelli et al. 2013). Importantly, in the present study frontal cortical structures were not found among clusters of significantly stronger activity in individuals with ASD.

In conclusion, we propose that the specific difficulties observed in the ASD group in attributing personal traits to the past self resulted in extensive engagement of neural mechanisms related mostly to the semantic and/or episodic components of autobiographic memory (Crane and Goddard 2008; Brezis 2015), with additional components of imagery (e.g. Addis et al. 2004) and embodied representations of the past events and facts (Pelphrey et al. 2011).

Finally, we would like to comment on the limitations of the present study. First of all, the study is confined by a small sample size. Thus, the reported results should be treated with caution and they need to be further investigated in larger groups of individuals with ASD in order to enhance statistical power. Secondly the experimental group was limited to high functioning adult males. Therefore our behavioral and brain imaging results could not be generalized to all individuals with ASD, irrespective of sex. Moreover, as they were high functioning individuals, there is a possibility that the alternative strategies of activation of brain areas that they showed in this study would be different from those detectable in low functioning persons with autism. In addition, a higher number of experimental trials/events focused on mechanisms of past self-processing would also be beneficial; this would enable more detailed investigation of effects related to—for instance—positive and negative adjectives that could be detected in fMRI data. Moreover, it would be worth to include present and past aspects of the close-other into experimental design of future studies in this field. We propose that in such case the close-other should be pre-defined as a friend that had been the friend since the time of attending a high school. As a consequence, the definition of past self and past close-other would be the same and participants would be required to refer to their and the close-other’s personal characteristics at present and during the same time period in their past. This approach would enable comparisons that may reveal additional inter-group differences associated with processing of information about the distant past.

## Abbreviations

ACG: Anterior cingulate gyrus
ASD: Autism spectrum disorder group
fMRI: Functional magnetic resonance imaging
IFG: Inferior frontal gyrus
l: Left
MCG: Middle cingulate gyrus
MFG: Middle frontal gyrus
MTG: Middle temporal gyrus
p: Posterior
PCG: Posterior cingulate gyrus
r: Right
STG: Superior temporal gyrus
TD: Typically developing group
TPJ: Temporoparietal junction
